# A Novel Follow-Up Model for Type 1 Diabetes in Children Leads to Higher Glycemic Control

**DOI:** 10.1155/pedi/6920068

**Published:** 2025-01-07

**Authors:** Julia Vonasek, Isabelle M. Larsen, Amar Nikontovic, Camilla Maria Thorvig

**Affiliations:** ^1^Pediatric Department, North Denmark Regional Hospital, Hjørring, Denmark; ^2^Mech-Sense, Department of Gastroenterology and Hepatology, Aalborg University Hospital, Aalborg, Denmark; ^3^Centre for Clinical Research, North Denmark Regional Hospital, Hjørring, Denmark; ^4^Steno Diabetes Center North Denmark, Aalborg University Hospital, Aalborg, Denmark

**Keywords:** glycemic control, HbA_1c_, pediatric diabetes, poorly regulated diabetes, quality improvement, type 1 diabetes

## Abstract

**Background:** Poor glycemic control in type 1 diabetes (T1D) in children leads to a higher risk of diabetic complications. In the pediatric department at the North Denmark Regional Hospital, only one-third of all children with diabetes were well-regulated, defined as HbA_1c_ no more than 58 mmol/mol (7.5%), in 2016. Therefore, a novel follow-up model was developed to increase the proportion of children with well-regulated T1Ds. The aim of this study was to evaluate the effect of a standardized follow-up model for poorly regulated diabetes on mean HbA_1c_.

**Methods:** All children aged 0–18 with T1Ds were included in this study. A novel standardized follow-up model was developed if HbA_1c_ was greater than 58 mmol/mol (7.5%), in which children were followed more closely until improvement in glycemic control.

**Results:** In the reference year, only one-third of children with diabetes were well-regulated and 19% were poorly regulated (HbA_1c_ greater than 75 mmol/mol (9.0%)). After fully implementing the model, two-thirds of the children had well-regulated diabetes, and only a few percent had poorly regulated diabetes. The mean HbA_1c_ decreased by almost 10 mmol/mol (or 0.8%) from the reference year to the following years when the model was fully implemented.

**Conclusion:** This follow-up model for poorly regulated diabetes increased the fraction of children with well-regulated diabetes in our clinic and significantly decreased mean HbA_1c_.

## 1. Introduction

Type 1 diabetes (T1D) in childhood is one of the most common chronic diseases in childhood [1], with about 300 new cases every year in Denmark [2]. Glycemic control in diabetes is important, as higher blood glucose levels correspond to a higher risk of known diabetic complications such as retinopathy, nephropathy, and neuropathy [3–7]. Furthermore, poor glycemic control correlates to a higher mortality rate [8]. HbA_1c_ is commonly used as an index for long-term glycemic control [9]. The glycemic control in children has improved during the last decades [10, 11]; however, there is still room for improvement. Studies have shown that greater involvement of the team around the child with T1D corresponds to a higher level of diabetic control [12–15]. We developed a new model to follow-up children with T1D in our outpatient clinic. The aim of this study was to evaluate the effect of this standardized follow-up model for children with poorly regulated T1D on mean HbA_1c_.

## 2. Methods

**Figure 1:**
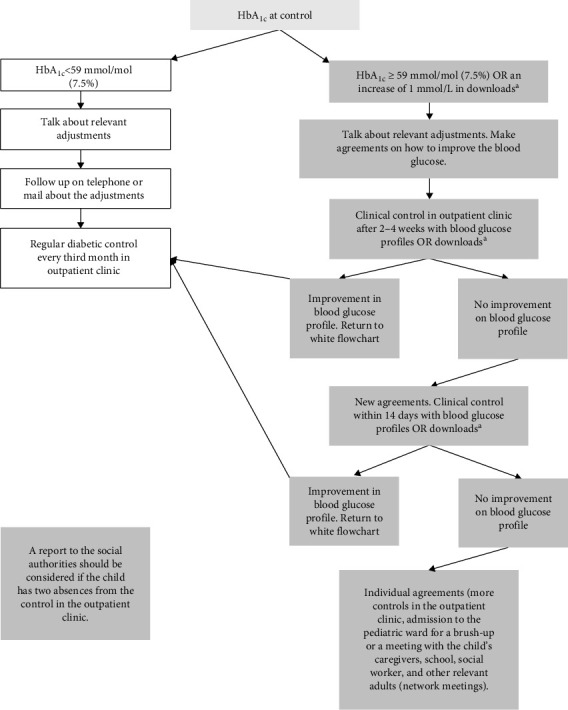
The novel follow-up model used in this article. Children with HbA_1 c_ < 59 (7.5%) remained on the left sided/gray arm until the next outpatient visit. Children with HbA_1 c_ ≥ 59 (7.5%) or an increase of 1 mmol/L in blood glucose profiles were followed in the right-sided arm until improvement. If still no improvement, further steps could be taken to regulate the HbA_1 c_. Two absences from the outpatient clinic should lead to a report to the social authorities. *Note:*^a^From the continuous glucose monitor.

Before July 1^st^, 2017, the offer for children with T1D in the pediatric department of the North Denmark Regional Hospital was the same for all children, with an outpatient appointment with blood samples every third month. There were some possibilities for an appointment with a general outpatient nurse for children with very poorly regulated diabetes. Telephone consultation was also available, offering the possibility of calling a diabetes nurse between 8 and 9a.m. every weekday. However, there was no standardized way of managing poorly regulated diabetes. From July 1^st^ 2017, a new standardized offer was implemented for children with T1D (see Figure 1). If a child's HbA_1c_ was greater than 58 mmol/mol (7.5%) or the blood glucose profile had increased by 1 mmol/L at the control visit in the outpatient clinic, the child was followed more closely until an improvement in blood glucose profiles was achieved. Blood glucose profiles were downloaded from the child's continuous glucose monitor and/or hybrid closed-loop devices. In our center, all children already had continuous glucose monitors and/or hybrid closed-loop devices in our reference year. The offer was fully implemented from January 1^st^, 2018. The new offer demanded additional staffing resources compared to the previous method of managing T1D. The aim was that more than 40% should have an HbA_1c_ of no more than 58 mmol/mol (7.5%) and no more than 20% of children with an HbA_1c_ greater than 75 mmol/mol (9.0%). For children with poorly regulated T1D and children who could not reach the target or uphold the agreements, there was the possibility of further increasing the effort to regulate HbA_1c_. These children and their families were offered admission to the pediatric department for a brush-up of the agreements. Furthermore, cooperation with social authorities and schools was established in the model and network meetings were offered. Children could also be referred for consultations with a psychologist to regulate HbA_1c_. A full description of the follow-up model can be found in the supplementary material. All agreements were recorded in the patient's electronic journal. We included all children aged 0–18 years with T1D who were followed in the pediatric department of the North Denmark Regional Hospital, Hjoerring, between 2016 and 2020. All consultations and HbA_1c_ values measured within the first 12 months after diagnosis were truncated to adjust for the confounding of the honeymoon period of T1D. Children were continuously included when diagnosed and excluded the day they turned 18. All HbA_1c_ values are indicated both in mmol/mol and (percentage). The clinical booking system (BookPlan) was used to find all contacts with the child and/or parents each year (telephone, email, letters, outpatient clinic appointments, psychologist consultations and network meetings). HbA_1c_ was identified in the clinical laboratory information system (LABKA II), while the date of diagnosis was found in the electronic health record system (Clinical Suite). Data were linked to the social security number [16] through the Patient Administrative System (PAS, AS400) in the North Denmark Region. STATA SE version 13 was used for data management and statistical analysis. We used the first year from July 2016 to 30 June 2017 (2016/2017) as the reference year and compared the three subsequent years (2017/2018, 2018/2019, and 2019/2020) to see the effect of the new follow-up model. A well-regulated T1D was defined as an HbA_1 c_ of no more than 58 mmol/mol (7.5%), moderately regulated between 59 and 75 mmol/mol (7.5–9.0%) and poorly regulated above 75mmol/mol (9.0%). These HbA_1 c_ limits were decided by the Danish Pediatric Diabetes Society (DanDiabKids), as limits for all pediatric departments in Denmark. The yearly mean HbA_1 c_ of each individual was used to compare the development. Quantile–quantile (QQ) plots were used to test for normal distribution. The HbA_1 c_ values were normally distributed and therefore mean and, to test for significance, the student's *t*-test was used. A *p*-value < 0.05 was considered significant. The age at diagnosis was normally distributed. All types of consultations were not normally distributed, and therefore the median, range, and interquartile range (IQR) were used to describe the data. A multivariable regression analysis was performed to test if sex and age had an independent influence on HbA_1 c_.

## 3. Results

For each year, there were between 73 and 80 children in our department, with an average age at diagnosis of 7.4–7.9 years. There were slightly more males than females. The mean HbA_1 c_ for all children was 65.6 mmol/mol (8.2%) in the reference year. Subsequently, it decreased to 58.4 mmol/mol (7.5%), and further to 56.9 mmol/mol (7.4%) in the following years, reflecting a statistically significant decrease (all p-values < 0.05) (Table 1). For the reference year, 36% of the children had well-regulated T1D, 45% moderately regulated T1D, and 19% poorly regulated T1D. After the implementation of the new follow-up model, the proportion of children with well-regulated T1D increased to 52%–67%. Almost two-thirds of the children in the reference year had moderately regulated T1D or poorly regulated T1D. For the last 2 years, when the follow-up program was fully implemented, nearly two-thirds of the children had well-regulated (65%–67%) T1D (Table 1). The proportion of consultations in the outpatient clinic in each age group remained stable during all 4 years (Table 1). The proportion of age groups also remained stable. As expected, the median number of consultations per child increased most among children with poorly regulated T1D. The number of all forms of contact with the department increased. The total number of outpatient consultations increased by 14% in 2017/2018, 38% in 2018/2019% and 12% in 2019/2020 compared to 2016/2017 (Table 2). However, the median number of consultations and IQR did not differ clinically significantly between years. Therefore, it is obvious that there are some children who had many consultations in an attempt to better regulate their T1D. Furthermore, there were only a few children who needed consultations with the psychologist and network meetings, to regulate their HbA_1 c_. The children with poorly regulated T1D were all in their teenage years (Table 1). In the multivariable regression analysis, age was as expected a significant factor for HbA_1c_-levels while sex was not. However, this did not differ between the years. From having no brush-up admissions in the pediatric department in the reference year, the number of brush-up admissions was three in 2017/2018, two in 2018/2019, and seven in 2019/2020 (Table 2). These admissions were among nine children, all among 13–17 years old children and lasted between 2 and 4 days.

## 4. Discussion

This study showed that the new follow-up program for all children with T1D had an effect on both mean HbA_1c_ and the proportion of children with well-regulated T1D. As the study was an open cohort study, each year had slightly different children as a child was included in the analysis 12 months after diagnosis and excluded when they were transferred to the adult clinic at 18 years of age. Therefore, it was not possible to perform a paired *t*-test to see if there was statistical significance in the proportion of children with well-regulated, moderately regulated, and poorly regulated T1D over the years. However, the result is of clinical significance as two-thirds had moderately regulated or poorly regulated T1D in the reference year and two-thirds had well-regulated T1D in the 2 years when the offer was fully implemented. The new follow-up program increased the activity of the diabetes team and therefore their workload. The program required two specially educated diabetes nurses for 32 h/week each, more workload for pediatricians and other employees in the pediatric department, and therefore increasing the budget for children with diabetes was necessary to maintain the offer. In the last year, 2019/2020, activity and the number of consultations decreased. However, the yearly mean HbA_1c_ and the proportion of children with well-regulated T1D were quite stable. This could be explained that the children and their families were now used to the new program and know that if they present in the clinic with an increase in HbA_1c_, the diabetes team will demand more controls; therefore, the children could be more prone to stay on track. This could also be explained by the COVID-19 pandemic and the lockdown of society. On March 11^th^ 2020, the Danish government introduced a strict lockdown due to the COVID-19 pandemic. Therefore, all planned nonlife-threatening consultations both at the general physician and in the hospital were canceled, and the number of consultations decreased in this period. The outpatient clinic reopened in mid-April and was back at normal level of consultations on the first of May. However, from 11th of March until the end of the last period, the society in Denmark was different than before March 11. The lower number of consultations in the last period did not, as mentioned above, result in an increase in HbA_1c_ or the proportion of children with poorly regulated T1D. However, since the yearly mean HbA_1c_ reported to the registry was used, the real effect on HbA_1c_ during the pandemic could be hidden by all HbA_1c_ taken before the outbreak. The pediatric department at the North Denmark Region Hospital was ranked ninth in the field of pediatric diabetes in Denmark before the implementation of this project [17]. In 2021 after its implementation, the department received the award for “best treatment of pediatric diabetes” in Denmark [18, 19]. The department was also awarded this prize in 2022 [20]. Therefore, the model has been a success for our department and the children and their families. Other diabetes centers have found an effect on HbA_1 c_ of increased involvement from the diabetes team [12–15]. The study by Doger et al. [12] only followed the children for 6 months, and it was always the parents or child that should contact the team, and not the team contacting the child/parents as in our model. They found an effect on HbA_1c_ in the group with the most contacts. Luca et al. [14] found an effect on HbA_1c_ during the time the intervention with coaching visits at home lasted. After the trial ended, HbA_1 c_ levels returned to a priori levels. The only study that resembled our study was conducted by Gandrud et al. [15]. Here, they compared regular follow-up visits (every third month) with an intervention group. In the intervention group, the team reviewed weekly uploaded blood glucose data and sent recommendations for tighter regulation of blood glucose to the family. The team found a trend toward better glycemic control in the intervention group. The best effect was found in the age group 13–17 years. 3 months after stopping the intervention, the effect was still present; however, the effect in the age group 13–17 years diminished. The studies mentioned above emphasize that greater team involvement is effective and the importance of maintaining the intervention to achieve lasting reduction in HbA_1c_. Hanberger et al. [21] enlightened the importance of structure and team cooperation in order to regulate HbA_1c_. Samuelsson et al. [11] evaluated a model to improve diabetes care in Sweden by educating the medical staff at the diabetes centers treating T1D in children. Here, they found an effect on HbA_1c_ in the participating centers. We have not found any studies that evaluated a model like ours. Studies have shown that HbA_1c_ tends to be higher in adolescents, especially in the late teenage years [22]. This age group is also overrepresented in the poorly regulated category in this study. However, the fraction of children with poorly regulated T1D decreased with our follow-up program, and therefore, this model also improves glycemic control in this age group.

This study has several limitations. There were problems with registration of email correspondences in the period 2018/2019; therefore, the real number of emails in this period is much lower than reported here. We do not know the exact number of email correspondences for this year. Another limitation of the study is that we have an open cohort. Furthermore, this model was not developed as a research project but as a quality improvement model due to the high number of children with poorly regulated T1D in our department. Therefore, our statistical power is low, and data that could be relevant, such as socioeconomic status, BMI and ethnicity, was not possible to obtain. In a quality improvement project, we only have access to some data about the child and their diabetes and it is not possible to divide the groups into an intervention group and a regular treatment group. In a quality improvement project, there is no different medication, way of taking medication, or other clinical interventions. These projects only concern the “way of handling” the disease in hospital settings. There are also limitations with the external validity of the study. In Denmark, healthcare is free of charge to all citizens and none of our children with diabetes lived more than 1 h away from the hospital. Therefore, in other countries where the distance to the hospital or healthcare system differentiates from our setup, our model might not be applicable. A telehealth approach might solve some of the issues with distance but was not tested in our setup due to the short distance to the hospital for the families and the possibility to schedule consultations until 6 PM. Therefore, telehealth is not warranted by our families. Also, our sample size is small. Greater prospective studies on the same population are warranted.

In conclusion, our novel follow-up model, in which children with T1D were stratified into different follow-up arms based on their quarterly HbA_1c_ levels, was effective and improved glycemic control for all age groups. The novel model required more resources from the diabetes team, and the number of contacts with the team increased.

## Figures and Tables

**Table 1:** Demographics, mean HbA_1 c_, HbA_1 c_ group, age group, and number of consultations by age and HbA_1 c_ group.

Year	2016/2017	2017/2018	2018/2019	2019/2020
Persons *n* (%)				
Total Male Female	80 41 (51) 39 (49)	73 39 (53) 34 (47)	75 43 (57) 32 (43)	75 43 (57) 32 (43)
Age group *n* (%)				
1–12 13–17	21 (26) 59 (74)	21 (29) 52 (71)	20 (27) 55 (73)	22 (29) 54 (71)
Age at diagnosis (years)				
Mean 95%CI	7.8 1.4–14.3	7.4 1.3−14−3	7.9 1.8–14.3	7.8 1.8–14.6
HbA_1 c_ (mmol/mol)				
mean 95%CI IQR *p*-value	65.6 47.9–90.5 56.0–71.2 ref	58.4 44.0–73.1 51.7–65.0 0.003	56.9 42.4–74.0 50.7–61.6 ≤0.001	57.7 40.3–79.6 51.2–62.2 0.001
HbA_1 c_ group *n* (%)				
<59 mmol/mol (<7.5%) 59–75 mmol/mol (7.5–9.0%) >75 mmol/mol (>9%)	29 (36) 36 (45) 15 (19)	38 (52) 32 (44) 3 (4)	50 (67) 23 (31) 2 (3)	49 (65) 21 (28) 5 (7)
Number of consultations in outpatient clinic by age group *n* (%)				
total 1–12 13–17	391 93 (24) 298 (76)	435 106 (24) 329 (76)	525 110 (27) 415 (73)	427 98 (23) 329 (77)
HbA_1 c_ group by age group *n* (% of total persons followed)				
<59 mmol/mol (<7.5%) 1–12 13–17 59–75 mmol/mol (7.5–9.0%) 1–12 13–17 >75 mmol/mol (>9%) 1–12 13–17	11 (14) 18 (23) 9 (11) 27 (34) 1 (1) 14 (18)	16 (22) 22 (30) 5 (7) 27 (37) 0 (0) 3 (4)	18 (24) 32 (43) 2 (3) 21 (28) 0 (0) 2 (3)	18 (24) 31 (41) 3 (4) 18 (24) 0 (0) 5 (7)
Number of outpatient consultations by HbA_1 c_ group *n* (% of total consultations) [median number of consultations per child]				
<59 mmol/mol (<7.5%) 59–75 mmol/mol (7.5–9.0%) >75 mmol/mol (>9%)	125 (32) [3] 199 (51) [4] 67 (17) [3]	197 (45) [4] 222 (51) [6] 16 (4) [3]	296 (56) [4] 199 (38) [6] 30 (6) [14]	214 (50) [3] 167 (39) [7] 42 (10) [7]

*Note:* Year 2016/2017 (1^st^ of July 2016 to 30^th^ June 2017) was used as a reference year.

**Table 2:** Number of consultations in outpatient clinic, mail, letter, telephone, network meetings, consultations with the psychologist, and brush-up admissions in the different years.

Year	2016/2017	2017/2018	2018/2019	2019/2020
Consultations outpatient clinic				
Total *n* Median (range) IQR	391 4 (0–11) 4–6	435 6 (0–15) 5–7	525 5 (1–32) 4–9	427 4 (1–19) 4–7
Telephone consultations				
Total *n* Median (range) IQR	239 2 (0–21) 1–4	397 4 (0–21) 2–9	675 6 (0–37) 3–15	369 4 (0–22) 2–7
Email consultations				
Total *n* Median (range) IQR	27 0 (0–3) 0–0	108 1 (0–7) 0–3	293 3 (0–20) 1–4	22 0 (0–2) 0–0
Letters				
Total *n* Median (range) IQR	74 1 (0–5) 0–1	33 0 (0–2) 0–1	21 0 (0–3) 0–0	20 0 (0–3) 0–0
Consultations with psychologist				
Total *n* Median (range) IQR	69 0 (0–12) 0–0	77 0 (0–15) 0–0	80 0 (0–14) 0–0	77 0 (0–14) 0–0
Meetings				
Total *n* Median (range) IQR	0 0 (0–0) 0–0	9 0 (0–2) 0–0	19 0 (0–4) 0–0	8 0 (0–2) 0–0
Brush-up admissions				
Total *n* Median (range) IQR	0 0 (0–0) 0–0	3 0 (0–2) 0–0	2 0 (0–2) 0–0	7 0 (0–2) 0–0

## Data Availability

The data are only available for this research group because it contains social security numbers.

## Supporting Information

Supporting Information In the file, a full description of the aim, target population and how the new model differentiated from the old model can be found.
