# Supplementary material for: A Novel Follow-Up Model for Type 1 Diabetes in Children Leads to Higher Glycemic Control
Source: Pediatr Diabetes. 2025 Jan 7;2025:6920068. doi: 10.1155/pedi/6920068 (PMC12017064; doi:10.1155/pedi/6920068)
Supplement: Supporting Information — In the file, a full description of the aim, target population and how the new model differentiated from the old model can be found. [file 6920068.f1.docx]

**Supplementary material: Full description of the new follow-up model**

**The aim of implementing a new follow-up model**

The aim of this initiative is to optimize a comprehensive treatment program for children with poorly regulated type 1 diabetes. The program is intended for children, adolescents, and their families.

**Rationale for implementing a new follow-up model**

As part of the agenda for the regional audit on diabetes care, the outpatient clinic at the pediatric department of the North Denmark Regional Hospital in Hjoerring was evaluated. This evaluation was prompted by several years of unsatisfactory results, where we were unable to meet key performance indicators.

One crucial indicator is that 40% of children should have well-regulated diabetes with an HbA_1c_ level no more than 58 mmol/mol (7.5%). In the 2016/2017 annual report, only 31% of children met this target. This indicates that the majority of children during this period had elevated long-term blood glucose levels. Another important indicator relates to children with very high long-term blood glucose levels above 75mmol/mol (9.0%). The target for this category is that no more than 20% of children should fall into this group. In the report, however, we had 21%, meaning that one in five children had a significantly elevated average blood glucose level and were at increased risk of developing long-term complications.

In order to prevent both acute and long-term complications, it is critical that children achieve well-regulated diabetes, ideally with an HbA_1c_ level no more than 58 mmol/mol (7.5%). The outpatient clinic experienced significant challenges in achieving the target of 40% of children with an HbA_1c_ < 59mmol/mol (7.5%).

**Target population**

Children with type 1 diabetes aged 0–18 years. All children referred to the outpatient clinic at the pediatric department of the North Denmark Regional Hospital in Hjoerring were included in the program.

**Detailed description of the new follow-up model**

**The diabetes outpatient clinic before the implementing the new model**

The diabetes team consisted of two outpatient nurses, each dedicating 10-15 hours per week to diabetes care. In addition, there were two senior consultants, one resident doctor, a dietitian, a psychologist, and three diabetes nurses within the department.

Prior to 1/7 2017, the program for children, adolescents, and their families involved four annual visits to the outpatient clinic, each with participation from a doctor and a nurse. For those with the greatest need, additional nursing consultations were offered between these visits. These additional consultations were primarily offered to patients with the most dysregulated diabetes or those requiring additional education. However, not all families could be offered this extra support.

Families were able to contact the diabetes nurses during scheduled phone hours between 8:00 and 9:00 AM. In addition, telephone consultations were available as needed, with the nurse initiating calls to the families. However, follow-up consultations were not consistently provided to all families.

Newly diagnosed children and their families received diabetes education from the clinic’s nurses and had the opportunity to meet with the outpatient nurses during hospitalization. During the first visit to the outpatient clinic, approximately one month after discharge, the family met with the outpatient nurse.

Network meetings were infrequently held.

Network meetings are collaborative meetings where the various professionals and support persons around the child come together. These meetings typically involve the parents, foster parents, teachers, school principals, social workers, and other relevant individuals involved in the child’s care.

The goal of these network meetings is to develop a comprehensive plan for how to best support the child and to gain a deeper understanding of how diabetes affects the child’s daily life.

**The diabetes outpatient clinic after implementing the new model**

From 1/7 2017 (or fully implemented from January 2018), the new follow-up model to improve the care provided to children and adolescents with diabetes was initiated.

The number of diabetes nurses was increased to two nurses working 32 hours per week. One of the nurses had no prior experience with diabetes and required training in diabetes care.

A flowchart was developed to better structure the follow-up and intervention process for families (figure 1 in the main article)

A letter outlining the new structure of the diabetes program was sent to families, and a user meeting was held to inform them about the new initiatives and improved opportunities for follow-up care.

The goals of the program were clarified: HbA_1c_ levels no more than 58 mmol/mol (7.5%). Depending on the individual needs of the family, intermediate goals were set.

Discussions were held with both the child and the family regarding how we could collaboratively work to achieve the established goal. If the goal was not met, agreements were made with families to submit data after 14 days, including readings from their continuous glucose monitor and/or hybrid closed loop devices. Follow-up visits in the outpatient clinic were scheduled for 7-14 days later, with new agreements established.

Families were contacted if they did not comply too the agreed-upon action plan, to understand the reasons and establish new agreements.

Follow-up via phone and email was conducted regularly.

An important element of the new model was the introduction of a primary nurse. Families were assigned a dedicated nurse who was familiar with the child, the family, and their resources. Based on the child’s and family’s available resources, an individualized care plan was developed using the flowchart. Some children benefited from weekly visits over a period of time, while others received sufficient support through bi-weekly phone calls. The assigned nurse was able to monitor the child’s progress continuously and intervene promptly, if necessary, through referral to psychologist or network meetings to prevent negative developments. Also, there was the possibility to refer the child to a brush-up admission at the department. A brush-up admission lasted for about three days. Here the diabetes nurses helped the child regain good glycemic control by education, help with insulin administration, carbohydrate counting and so forth to help the child master the disease when they got discharged again.

An additional change in the new model was that diabetes nurses in the outpatient clinic were tasked with educating newly diagnosed children, adolescents, and their families about diabetes care. This allowed the team to get to know the family and their resources from the outset.

An increase in HbA_1c_ was followed up with additional visits, phone calls, and HbA_1c_ measurements between regular consultations, as well as additional downloads from devices.

Faster interventions, including referrals to local social services and network meetings, were triggered if psychosocial factors were identified as contributing to the dysregulation of the child’s diabetes management.

Ongoing education was offered to teachers and daycare staff, ensuring they were better equipped to support children with diabetes in their everyday environments.
